# Integrated analysis of immune parameters, miRNA-mRNA interaction, and immune genes expression in the liver of rainbow trout following infectious hematopoietic necrosis virus infection

**DOI:** 10.3389/fimmu.2022.970321

**Published:** 2022-09-02

**Authors:** Shenji Wu, Jinqiang Huang, Yongjuan Li, Mingquan Lei, Lu Zhao, Zhe Liu

**Affiliations:** ^1^ College of Animal Science and Technology, Gansu Agricultural University, Lanzhou, China; ^2^ College of Science, Gansu Agricultural University, Lanzhou, China

**Keywords:** rainbow trout, IHNV, immune parameters, RNA-seq, expression analysis, functional analysis

## Abstract

Rainbow trout (*Oncorhynchus mykiss*) is an important economical cold-water fish worldwide. However, infection with infectious hematopoietic necrosis virus (IHNV) has severely restricted the development of aquaculture and caused huge economic losses. Currently, little is known about the immune defense mechanisms of rainbow trout against IHNV. In this study, we detected the changes of immune parameters over different post-infection periods (6-, 12-, 24-, 48-, 72-, 96-, 120-, and 144 hours post-infection (hpi)), mRNA and miRNA expression profiles under 48 hpi (T48L) compared to control (C48L), and key immune-related genes expression patterns in rainbow trout liver following IHNV challenge through biochemical methods, RNA-seq, and qRT-PCR, and the function of miR-330-y was verified by overexpression and silencing *in vitro* and *in vivo*. The results revealed that alkaline phosphatase (AKP), alanine aminotransferase (ALT), catalase (CAT), and total superoxide dismutase (T-SOD) activities, and lysozyme (LZM) content showed significant peaks at 48 hpi, whereas malondialdehyde (MDA) content and aspartate aminotransferase (AST) activity decreased continuously during infection, and acid phosphatase (ACP) activity varied slightly. From RNA-seq, a total of 6844 genes and 86 miRNAs were differentially expressed, and numerous immune-related differentially expressed genes (DEGs) involved in RIG-I-like receptor signaling pathway, Toll-like receptor signaling pathway, NOD-like receptor signaling pathway, cytokine-cytokine receptor interaction, and antigen processing and presentation were significantly upregulated in T48Lm group, including *IFIH1*, *DHX58*, *MAVS*, *TRAF3*, *IRF3*, *IRF7*, *MX1*, *TLR3*, *TLR8*, *MYD88, NOD1*, *NOD2*, *IL-8*, *CXCR1*, *CD209*, *CD83*, and *TAP1*. Integrated analysis identified seven miRNAs (miR-425-x, miR-185-x, miR-338-x, miR-330-y, miR-361-x, miR-505-y, and miR-191-x) that target at least three key immune-related DEGs. Expression analysis showed that *IFIH1*, *DHX58*, *IRF3*, *IRF7*, *MX1*, *TLR3*, *TLR8*, and *MYD88* showed a marked increase after 24 hpi during infection. Further research confirmed *TAP1* as one of the targets of miR-330-y, overexpression of miR-330-y with mimics or agomir significantly reduced the expression levels of *TAP1*, *IRF3*, and *IFN*, and the opposite effects were obtained by inhibitor. These results facilitate in-depth understanding of the immune mechanisms in rainbow trout against IHNV.

## Introduction

Aquaculture is a growing agribusiness that improves food quality and drives economic development of the country. Rainbow trout (*Oncorhynchus mykiss*), a member of the salmonid fish, is one of the most commercially important cold-water fish worldwide. This species is farmed in more than seventy countries with global production around 800 thousand tonnes (FAO, 2020). A pivotal challenge for this industry is to meeting the needs of increased consumption for rainbow trout. Under intensive conditions of aquaculture, the cultured fish must live in a complex aquatic environment containing various non-pathogenic and pathogenic hazards, resulting in increased susceptibility of disease with pathogens load (1). Infectious hematopoietic necrosis (IHN), a highly contagious viral disease, may cause up to 100% mortality for fry and juvenile rainbow trout during outbreaks caused by IHN virus (IHNV; family *Rhabdoviridae*, genus *Novirhabdovirus*), which is now spread throughout Canada, Europe, Japan, and China since the first reported in the United States, and has posed severe economic losses for aquaculture production (2–4). At present, there is no way to completely stop the spread of IHN, including vaccines. Thus, selective breeding of rainbow trout strains with genetic resistance to IHNV is warranted.

The innate immune system provides a first line of defense that recognizes, responds to, and eliminates invading pathogens, and immune-related genes have been considered as critical drivers of the initiation and progression of pathogen infection (5). High-throughput sequencing is an efficient technology to systemically examine patterns of transcription expression in tissues of interest. Over the past few years, there have been numerous reports that immune-related molecules response to IHNV infection in various salmonid fish. In Sockeye salmon (*Oncorhynchus nerka*), expression levels of myxovirus resistance-1 (*MX1*), interleukin-1β (*IL-1β*), interleukin-8 (*IL-8*) were enhanced in the head kidney by IHNV challenge (6). The interferon (*IFN*), transcription factors (*IRF3*, *IRF7*) and signal transducer and activator of transcription 1 (*STAT1*) genes were highly expressed after IHNV infection in the head kidney of rainbow trout (7). Rainbow trout gonadal fibroblasts (RTG-2 cells) infected with IHNV exhibited elevated expression of virus-induced CXC chemokines and caspase 8 (*CASP8*) (8). Although these studies identified several key genes in salmonid fish against IHNV infection, the responses of numerous immune-regulated genes remain unclear.

MicroRNAs (miRNAs) are primarily defined as RNAs that are approximately 19–25 nucleotides in length but have no protein-coding capacity (9). Initially, they were regarded as the “transcriptional noise” without biological function. The emerging evidences showed that miRNAs emerged as critical component of host-virus interactions during the viral infection. Large amounts of fish miRNAs involved in the immune response related to pathogens infection have been identified. These miRNAs include the Atlantic salmon (*Salmo salar*) induced by infectious pancreatic necrosis virus (IPNV) (10), tilapia (*Oreochromisspp*) induced by tilapia lake virus (TiLV) (11), and Japanese flounder (*Paralichthys olivaceus*) induced by *Vibrio anguillarum* (12), which were mainly involved in RIG-I-like receptor (RLR) signaling pathway, Toll-like receptor (TLR) signaling pathway, NOD-like receptor (NLR) signaling pathway, Jak-STAT signaling pathway, and apoptosis. Moreover, Cao et al. (13) found that overexpression of miR-146a-3p and miR-216a-5p in RTG-2 cells of rainbow trout infected with IHNV significantly decreased the expression of *IFN* and *MX1*, and increased viral titers (terms of 50% tissue culture infectious dose, TCID_50_). These reports demonstrated that miRNAs with variable expression levels play the significant roles in fish immune responses.

The liver is a key metabolic and immune organ in fish, and critical metabolic functions often eclipse its role as an essential organ for immune regulation (14). As an immune organ, the liver contains various natural immune cells, which can produce cytokines, chemokines, complement components, and APR proteins, suggesting it performs a crucial role in response to virus invasion (15). In view of the particularity of the liver, it is necessary to understand the changes of immune parameters and miRNA-mRNA expression in the liver of rainbow trout after IHNV infection.

In this study, both biochemical methods and RNA-seq were performed to analyze the changes of immune parameters and miRNA-mRNA expression profiles in the rainbow trout liver against IHNV infection. The effects of IHNV on key immune parameters and genes were determined, and immune-related miRNA-mRNA pairs and the function of miR-330-y were also identified. The results contribute to our understanding of the immune mechanisms in rainbow trout against IHNV, and lay a foundation for further studying the regulatory functions of miRNAs on key immune-related genes.

## Materials and methods

### Experimental animals, IHNV challenge, and sample collection

Rainbow trout belonging to a full-sib family used in this study were obtained from Aquatic Science Training Center of Gansu Agricultural University in Gansu province, China. Fish with similar size (10.50 ± 0.50 g) were selected and maintained in a flow-through water system (3000 L) with temperature 12.00 ± 0.50 °C, dissolved oxygen > 8.50 mg/L, NH4-N < 0.10 mg/L for two weeks prior to the experiment. Fish were fed with commercial pellet feed (Tongwei, China) at 1.5% of body weight twice daily. For the challenge, the fish were injected intraperitoneally with 100 µL IHNV containing 500 plaque forming units (pfu) per individual (16, 17), and control fish were carried out the same treatments with 100 µL cell medium (Medium 199, HyClone, USA). After the fish treated with tricaine methanesulfonate (MS-222, Sigma, St. Louis, MO, USA) (400 mg/L), liver samples were taken at 6 hours post-infection (hpi), 12 hpi, 24 hpi, 48 hpi, 72 hpi, 96 hpi, 120 hpi, and 144 hpi, and immediately stored in liquid nitrogen. Each time point contained three replicates. All experiments complied with institutional guidelines and the protocol approved by the Animal Experimentation Ethics Committee at Gansu Agricultural University, China.

### Determination of immune parameters

To assess the effect of IHNV on rainbow trout immunity, various immune parameters of the liver over different post-infection periods (6-, 12-, 24-, 48-, 72-, 96-, 120-, and 144 hpi) were measured according to the manufacturer’s directions of analysis kits (Nanjing Jiancheng Bioengineering Institute, Nanjing, China) (n=3), including alkaline phosphatase (AKP), acid phosphatase (ACP), lysozyme (LZM), total superoxide dismutase (T-SOD), catalase (CAT), malondialdehyde (MDA), alanine aminotransferase (ALT), and aspartate aminotransferase (AST).

### Construction and sequencing of mRNA and miRNA libraries

Based on the results of immune parameters, the liver samples of control (C48L) and 48 hpi (T48L) were determined for mRNA and miRNA sequencing (n = 3 per group). Firstly, extraction of total RNA was performed using Trizol reagent kit (Invitrogen, Carlsbad, CA, USA), then the RNAs concentration and quality were respectively assessed by an Agilent 2100 Bioanalyzer (Agilent Technologies, Palo Alto, CA, USA) and RNase free agarose gel electrophoresis. Subsequently, the first-strand cDNA was synthesized with random primers and reverse transcriptase from purified mRNA, followed by second-strand cDNA synthesis with DNA polymerase I, RNase H, dNTP, and buffer. Finally, cDNA libraries were constructed and sequenced on the Illumina HiSeq2500 platform, with 150 bp pair-end reads produced.

For miRNA sequencing, a size range of 18–30 nt RNA bands were isolated by polyacrylamide gel electrophoresis, and 5′ and 3′ adapters were ligated. Then, the final PCR products were generated after reverse-transcription PCR and sequenced on the Illumina HiSeq2500 platform by single-end sequencing (50 bp).

### Raw data processing and identification of differentially expressed genes and miRNAs

Raw reads from mRNA-seq were filtered with FASTP (version 0.18.0) to obtain high-quality clean reads. Briefly, adaptor sequences, unpaired reads, and low-quality bases (Q < 20) were removed using FastQC software. HISAT2.2.4 was used to map pairs of terminal clean reads to the rainbow trout reference genome (Omyk_1.0). After the mapping, the expression level of each transcript was calculated based on fragment per kilobase of transcript per million mapped reads (FPKM). We used DESeq2 (v.1.6.3) to determine the false discovery rate (FDR) threshold, and |Log_2_ fold change | ≥ 1 and FDR < 0.05 were used as the criteria to define DEGs between C48Lm (control) and T48Lm (48 hpi) groups (18).

The clean miRNA reads were obtained from raw reads after discarding sequences, adaptor-ligated contaminants, and reads shorter than 18 nt. Clean reads were compared against small RNAs in Rfam database to identify and remove rRNA, scRNA, sonRNA, snRNA, and tRNA. Finally, known miRNAs were identified by aligning against the miRbase database (v.22), and unannotated reads were considered as novel miRNAs according to their genome positions and hairpin structures using Mireap_v0.2. The expression of known and novel miRNAs was normalized to transcripts per million (TPM) following formula: TPM = Actual miRNA counts/(Total miRNA counts) × 10^6^. DEMs between C48Ls (control) and T48Ls (48 hpi) groups were analyzed with DESeq2 (v.1.6.3) based on the following thresholds: |Log_2_ fold change | ≥ 1 and *p*value < 0.05 (18).

### Target genes prediction of DEMs and miRNA-mRNA regulatory network construction

The target genes of DEMs were predicted by RNAhybrid (v2.1.2) + svm_light (v6.01), Miranda (v3.3a), and TargetScan (Version: 7.0). To elucidate the possible miRNA–mRNA interaction, the expression correlation between miRNA and its target was assessed using the Pearson correlation coefficient (PCC). Pairs with PCC < −0.7 and *p*value < 0.05 were selected as co-expressed negatively miRNA-mRNA pairs, and all RNAs were differentially expressed. The concerned negative relationship of miRNA-mRNA pairs were screened out to construct the regulatory network with Cytoscape (v3.6.0).

### Functional enrichment of DEGs and targets of DEMs, and protein-protein interaction analyses

To assess functional enrichment, Gene Ontology (GO) and Kyoto Encyclopedia of Genes and Genomes (KEGG) analyses of DEGs and targets of DEMs were performed. Briefly, GO analysis consisted of three components: biological process, molecular function, and cellular component. KEGG analysis was carried out to help elucidate the biological functions and critical signal pathways of DEGs and targets of DEMs. The statistical enrichment of GO terms and KEGG pathway with *q*value < 0.05 were regarded as statistically significant. PPI network analysis was performed using STRING (https://string-db.org/).

### Validation of the RNAs sequencing data and analysis of key immune-related genes expression by qRT-PCR

To validate the reliability of the high-throughput sequencing data, qRT-PCR of 10 selected DEGs (interferon induced with helicase C domain 1 (*IFIH1*/*MDA5*), DEXH (Asp-Glu-X-His) box polypeptide 58 (*DHX58*/*LGP2*), mitochondrial antiviral signaling protein (*MAVS*/*IPS-1*), DEAD-box helicase 3 X-linked (*DDX3X*), TRAF family member-associated NFKB activator (*TANK*), Toll-like receptor 3 (*TLR3*), myeloid differentiation factor 88 (*MYD88*), *IRF3*, *IRF7*, and *MX1*) and 8 selected DEMs (miR-452-x, miR-151-y, miR-27-x, miR-363-y, miR-582-y, miR-339-x, miR-338-x, and miR-330-y) were conducted. The RNAs of the samples used for qRT-PCR were the same as those used for RNAs sequencing. Reverse transcription of mRNA and miRNA were performed using a PrimerScript RT Reagent Kit with gDNA Eraser (Takara, Dalian, China) and a Mir-X miRNA First-Strand Synthesis Kit (Clontech, Mountain View, CA, USA) following the manufacturer’s instructions. The *β-actin* and *U6* were respectively taken as the reference gene for normalizing mRNA and miRNA expression (19). Then, qRT-PCR with SYBR Premix Ex Taq (Takara) was executed on a LightCycler^®^ 480 II Instrument (Roche, Basel, Switzerland) according to the manufacturer’s protocol (n=3). PCR cycles were as follows: 95°C for 10 s, followed by 40 cycles at 95°C for 5 s and 60°C for 20 s. The specificities of the PCR products were determined by melting-curve. Besides control and 48 hpi, total RNA from the other seven time points were extracted using Trizol reagent kit (Invitrogen, Carlsbad, CA, USA) following the manufacturer’s instruction, including 6 hpi, 12 hpi, 24 hpi, 72 hpi, 96 hpi, 120 hpi, and 144 hpi. Expression trends of key immune-related genes selected from mRNA-seq at the above nine time points were analyzed by qRT-PCR (n=3). The sequences of all primers are shown in **Table S1**. The mRNAs and miRNAs expression levels relative to the reference gene were calculated using the 2^-ΔΔCt^ method. qRT-PCR data were presented as means ± SD and compared using one-way ANOVA followed by Dunn’s test in SPSS version 22.0 (IBM Corp, Armonk, NY, USA).

### Dual-luciferase reporter assay

Based on the results of miRNA-mRNA regulatory network, miR-330-y was predicted to target *TAP1* (XM_021559784.2). The pmirGLO-*TAP1*-3′untranslated region (UTR)-wild type plasmids and pmirGLO-*TAP1*-3′UTR-mutant plasmids (500 ng/μL) were constructed, and were co-transfected with miR-330-y mimics or negative control (NC) (20 μM) into HEK293 cells in 96-well plates using INVI DNA RNA transfection Rragent (Invigentech, USA) (n = 3). Luciferase assay was carried out at 48 h post-transfection through the Dual-Glo^®^ Luciferase Assay System (Promega, USA) following the manufacturer’s protocols, and relative activity of fluorescein (Firefly luciferase activities/Renilla luciferase activities) was obtained.

### Over-expression and silencing of miR-330-y *in vitro*

To study the effects of miR-330-y on the expression *TAP1* and its regulated gene (*IRF3* and *IFN*), rainbow trout liver cells were used for functional analysis of miR-330-y *in vitro*. Rainbow trout liver cells were cultured in dulbecco’s modified eagle medium (DMEM) high glucose cell medium containing 15% fetal bovine serum (Gibco, USA), 1% 400 IU/mL penicillin-400 IU/mL streptomycin-25 μg/mL amphotericin mixture, and 0.5% 4.5 M NaCl at 18°C in a 5% CO_2_ incubator. When density reached about 80% in 24-well plates, liver cells were transfected with miR-330-y mimics, miR-330-y inhibitor, and their NC (20 μM) (GenePharma, China) using INVI DNA RNA transfection Rragent (Invigentech, USA) according to the manufacturer’s direction (n = 3). After 48 h post-transfection, cells were collected and used for subsequent examination of the expressions of miR-330-y, *TAP1*, *IRF3*, and *IFN* using qRT-PCR method described above.

### Over-expression of miR-330-y *in vivo*

We then further used agomir to evaluate the effects of miR-330-y over-expression on the expression levels of *TAP1*, *IRF3*, and *IFN in vivo*. Eight rainbow trout (25.00 ± 0.50 g) with similar color from a full-sib family were selected and randomly distributed two groups named as miR-330-y agomir group and miR-330-y agomir NC group. Subsequently, fish were injected with miR-330-y agomir and miR-330-y agomir NC (GenePharma, China) *via* the tail vein for three consecutive days, respectively. Three days later, the fish livers were sampled and used for miR-330-y, *TAP1*, *IRF3*, and *IFN* expression analysis.

## Results

### Changes of immune parameters of liver infected with IHNV

**Figure 1:**
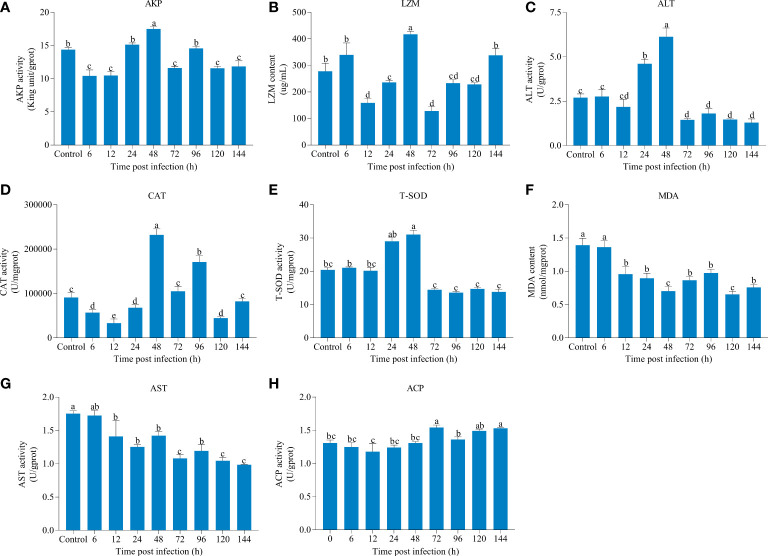
The changes of immune parameters of liver over different periods after infectious hematopoietic necrosis virus (IHNV) infection. **(A)** Alkaline phosphatase (AKP). **(B)** Lysozyme (LZM). **(C)** Alanine aminotransferase (ALT). **(D)** Catalase (CAT). **(E)** Total superoxide dismutase (T-SOD). **(F)** Malondialdehyde (MDA). **(G)** Aspartate aminotransferase (AST). **(H)** Acid phosphatase (ACP). The different lowercase letters above the bars represent significant differences (*P* < 0.05).

The effects of IHNV on liver immune parameters in rainbow trout were shown in **Figure 1**. As compared to the other eight time points, AKP, ALT, CAT, and T-SOD activities and LZM content reached its highest level at 48 hpi, and the activities of AKP, ALT, and T-SOD were decreased to varying degrees after 48 hpi compared with the control. Moreover, we found that AST activity and MDA content were lower than those of the control at each time point after infection, and there was a little change in the activity of ACP over different periods.

### Overview of mRNA and miRNA sequencing data

To better understand the immune mechanisms after IHNV infection, we conducted a comparative mRNA and miRNA expression profiles analysis between C48L and T48L groups. From the libraries of mRNA-seq, a total of 39,632,545,200 raw reads with an average of 6,605,424,200 reads for each sample were generated. All raw reads were submitted to the Gene Expression Omnibus (GEO) database at National Center for Biotechnology Information (NCBI) (GSE205742). The average of quality Q20 and Q30 were higher than 98.05% and 94.31% for each library, respectively, implying that the sequencing data were of high quality. Across six libraries, the GC content ranged from 50.91% to 51.76%. After filtering, 39,358,806,614 clean reads were obtained, and 86.36–87.76% for each library was mapped to the reference genome. Overviews of the specific sequencing and assembly results for C48Lm and T48Lm groups are shown in **Table 1**. Pearson’s correlation coefcients for sample expression were 97.26–98.24% for C48Lm-1, C48Lm-2, and C48Lm-3, 98.30–99.27% for T48Lm-1, T48Lm-2, and T48Lm-3, suggesting the reliability among these replicates and sequencing data produced in this study could be used for subsequent analysis (**Figure S1A**).

**Table 1:** Statistical results of comparison rate between reads and reference genomes.

Sample name	C48Lm-1	C48Lm-2	C48Lm-3	T48Lm-1	T48Lm-2	T48Lm-3
**Raw reads**	48,463,260	43,263,476	46,897,752	40,461,658	42,338,756	42,792,066
**Clean reads**	48,318,822	43,137,724	46,763,586	40,356,426	42,236,284	42,659,326
**Q20 (%)**	98.21%	98.26%	98.12%	98.19%	97.46%	98.08%
**Q30 (%)**	94.73%	94.86%	94.54%	94.65%	92.71%	94.42%
**GC content (%)**	51.11%	51.72%	51.76%	50.92%	51.06%	50.91%
**Total mapped**	42,301,599 (87.57%)	37,709,558 (87.43%)	40,377,718 (86.36%)	35,375,811 (87.68%)	37,026,559 (87.68%)	37,429,569 (87.76%)
**Multiple mapped**	3,789,757 (7.84%)	3,556,932 (8.25%)	3,849,203 (8.23%)	3,170,602 (7.86%)	3,344,646 (7.92%)	3,322,004 (7.79%)
**Uniquely mapped**	38511842 (79.72%)	34152626 (79.19%)	36528515 (78.13%)	32205209 (79.82%)	33681913 (79.76%)	34107565 (79.97%)

For six miRNA libraries, a total of 36,205,253 and 35,723,290 raw reads were acquired from C48Ls and T48Ls groups and deposited in the NCBI under the accession number GSE205742, and 35,571,875 and 35,119,539 high quality clean reads were generated after filtering the raw reads (**Table 2**). The total mapped tags to the reference genome in the C48Ls and T48Ls groups were 29,932,527 (90.43%) and 29,489,693 (91.20%), respectively. The length distribution of miRNA in all six libraries mainly concentrated in 21–23 nt, with 22 nt being the most abundant (**Figure S1B**). The first nucleotide bias showed that U was present in the largest proportion. Based on the comparison to reference genome sequence, 618 known miRNAs and 1408 novel miRNAs were identified in these six libraries (**Figure S1C**).

**Table 2:** Categorization of rainbow trout non-coding and organellar small RNAs.

Sample name	C48Ls-1	C48Ls-2	C48Ls-3	T48Ls-1	T48Ls-2	T48Ls-3
**Clean reads**	11,475,475	11,492,703	13,237,075	13,306,417	11,707,227	10,709,646
**High quality reads**	11,303,003	11,274,169	12,994,703	13,069,970	11,505,876	10,543,693
**Mapped reads**	9,675,544 (90.00%)	9,544,642 (91.63%)	10,712,341 (89.66%)	10,820,601 (91.31%)	9,755,915 (90.96%)	8,913,177 (91.34%)
**Exon**	82,298	79,559	82,436	99,625	103,603	115,269
**Known miRNA**	8,161,789 (75.92%)	7,158,595 (68.73%)	7,891,193 (66.05%)	8,190,412 (69.11%)	7,420,354 (69.19%)	6,308,354 (64.64%)
**Novel miRNA**	8574 (0.08%)	8481 (0.08%)	11,574 (0.10%)	10,542 (0.09%)	8588 (0.08%)	7665 (0.08%)
**rRNA**	2,047,123 (19.04%)	2,764,324 (26.54%)	3,363,854 (28.15%)	3,044,578 (25.69%)	2,730,668 (25.46%)	2,866,051 (29.37%)
**tRNA**	77,946 (0.72%)	63,080 (0.61%)	76,344 (0.64%)	65,207 (0.55%)	61,042 (0.57%)	64,394 (0.66%)
**snRNA**	20,239 (0.19%)	17,953 (0.17%)	25,794 (0.22%)	26,088 (0.22%)	21,151 (0.20%)	21,018 (0.22%)
**snoRNA**	24,276 (0.23%)	19,540 (0.19%)	32,231 (0.27%)	25,768 (0.22%)	21,064 (0.20%)	19,317 (0.20%)
**Others**	168,613 (1.57%)	181,119 (1.74%)	300,657 (2.52%)	217,608 (1.84%)	203,275 (1.90%)	200,313 (2.05%)

### Identification of DEGs and GO enrichment and KEGG pathway analyses

Concerning the mRNA sequencing data analysis, our project detected 40,307 genes, and the distribution of gene expression abundance was assessed for each sample (**Figure S1D**). Using a cutoff of |Log_2_ fold change | ≥ 1 and FDR < 0.05, 6844 DEGs were recognized between C48Lm and T48Lm groups, and 3316 DEGs were upregulated in T48Lm group, while 3528 DEGs were downregulated (**Table S2** and **Figure S2A**). Hierarchical clustering analysis of all DEGs was shown in **Figure S2B**. Among these DEGs, it was worth noting that many immune-related DEGs were significantly upregulated in T48Lm group, and detail information of their expression and hierarchical clustering between C48Lm and T48Lm groups are respectively shown in **Table 3** and **Figure S2C**. To further analyze the relationships among these immune-related DEGs, PPI network was constructed (**Figure 3D**).

**Table 3:** Expression and annotation of immune-related differentially expressed genes (DEGs) between C48Lm and T48Lm groups.

Gene	Annotation		C48Lm_FPKM	T48Lm_FPKM	Log_2_ (FC)	FDR
**RIG-I like receptor signaling pathway**						
*IFIH1*		Melanoma differentiation associated gene 5	5.77	45.82	2.99	1.32E-102
*DHX58*		DEXH (Asp-Glu-X-His) box polypeptide 58	0.92	188.76	7.68	8.20E-272
*MAVS*		Mitochondrial antiviral signaling	0.59	1.28	1.11	0.03
*TRAF3*		TNF receptor-associated factor 3	0.36	6.05	4.06	1.12E-39
*TANK*		TRAF associated NF-kappa-B activator	3.03	7.72	1.35	7.43E-08
*IKBKE*		Inhibitor of nuclear factor kappa-B kinase subunit epsilon	1.25	19.41	3.95	4.51E-64
*DDX3X*		DEAD-box helicase 3 X-linked	9.88	68.99	2.80	3.44E-72
*IRF3*		Interferon regulatory factor 3	2.01	37.75	4.23	2.23E-130
*IRF7*		Interferon regulatory factor 7	0.63	38.82	5.95	5.65E-127
*TRIM25*		E3 ubiquitin/ISG15 ligase TRIM25	8.99	40.53	2.17	2.11E-62
**Toll-like receptor signaling pathway**						
*TLR3*		Toll-like receptor 3	6.41	38.44	2.58	6.59E-82
*TLR8*		Toll-like receptor 8	0.01	0.16	3.97	2.50×10^-3^
*MYD88*		Myeloid differentiation factor 88	2.95	13.67	2.40	2.94E-24
*IRAK4*		Interleukin-1 receptor-associated kinase 4	2.13	5.56	1.39	5.91E-08
*IRAK1*		Interleukin-1 receptor-associated kinase 1	0.89	1.97	1.14	2.26×10^-3^
**NOD-like receptor signaling pathway**						
*NOD1*		Nucleotide-binding oligomerization domain protein 1	0.79	6.35	3.01	2.44E-47
*NOD2*		Nucleotide-binding oligomerization domain protein 2	0.36	2.53	2.82	7.36E-20
*RIPK2*		Receptor-interacting serine-protein kinase 2	0.28	1.07	1.96	3.30E-10
*NLRC3*		Nucleotide-oligomerization domain receptor C3	0.63	4.77	2.91	3.38E-40
*NLRC5*		Nucleotide-oligomerization domain receptor C5	0.17	7.14	5.34	8.49E-99
**Cytokine-cytokine receptor interaction**						
*IL-8*		Interleukin-8	0.25	1.40	2.48	1.50×10^-3^
*CXCL10*		C-X-C chemokine ligand 10	0.08	26.00	8.41	1.98E-21
*CXCL11*		C-X-C chemokine ligand 11	0.05	6.68	7.16	2.63E-10
*CCL19*		C-C chemokine ligand 19	0.01	11.69	13.51	7.86E-19
*CXCR1*		C-X-C chemokine receptor 1	0.09	1.95	4.49	1.90E-11
*CCR8*		C-C chemokine receptor type 8	0.55	1.63	1.56	4.62E-05
*CCR9*		C-C chemokine receptor type 9	0.47	1.53	1.69	0.03
**Antigen processing and presentation**						
*CD209*		C-type lectin domain family 4 member E	112.39	1937.21	4.11	2.94E-88
*CD83*		CD83 antigen	0.15	1.53	3.38	3.01E-09
*TAP1*		Antigen peptide transporter 1	6.17	88.68	3.85	6.46E-152
*TAP2*		Antigen peptide transporter 2	0.47	31.83	6.07	4.05E-141
*MHCI*		MHC class I alpha chain precursor	22.11	53.61	1.28	1.48E-19

**Figure 2:**
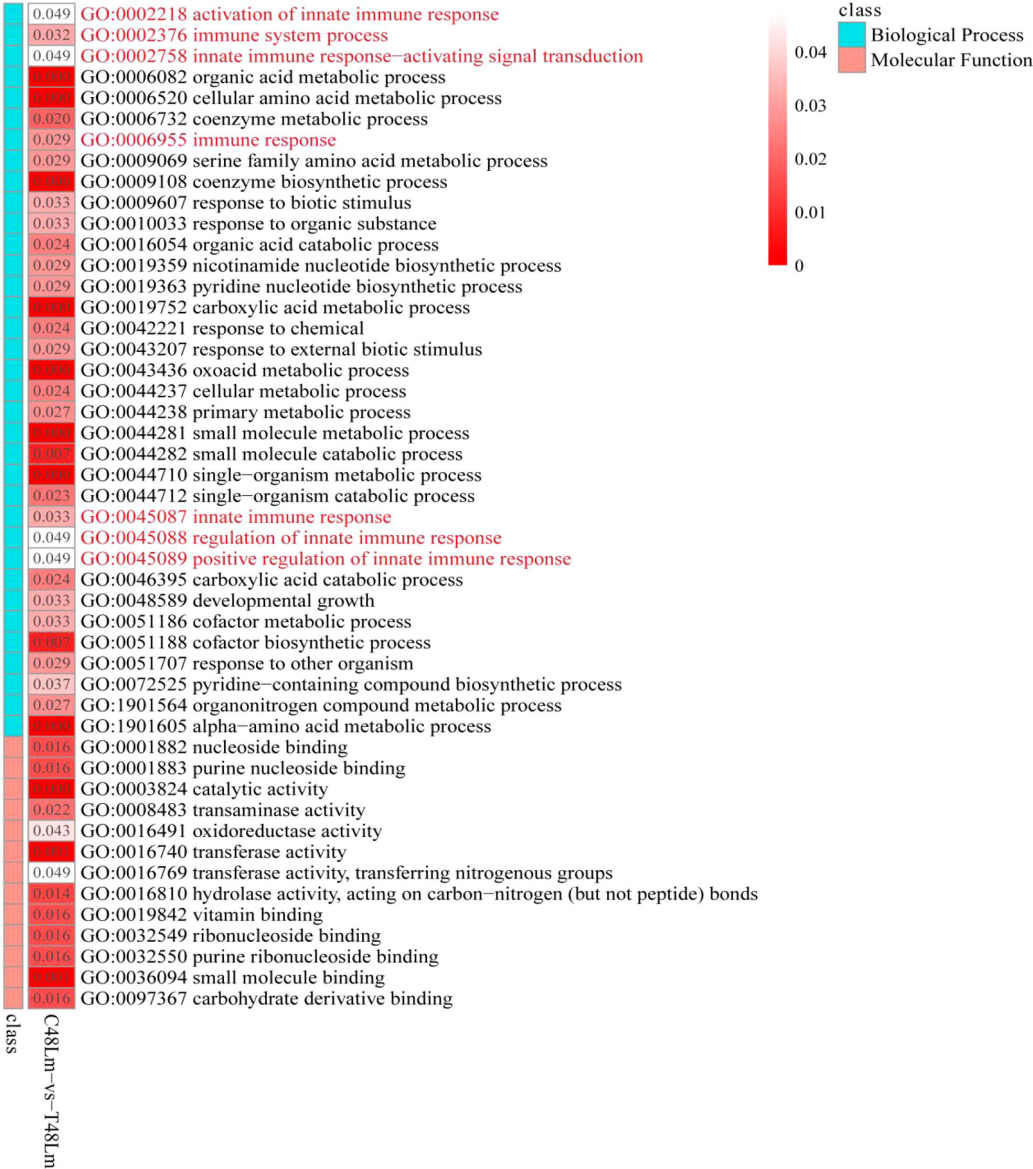
GO enrichment analysis of DEGs between C48Lm and T48Lm groups.

**Figure 3:**
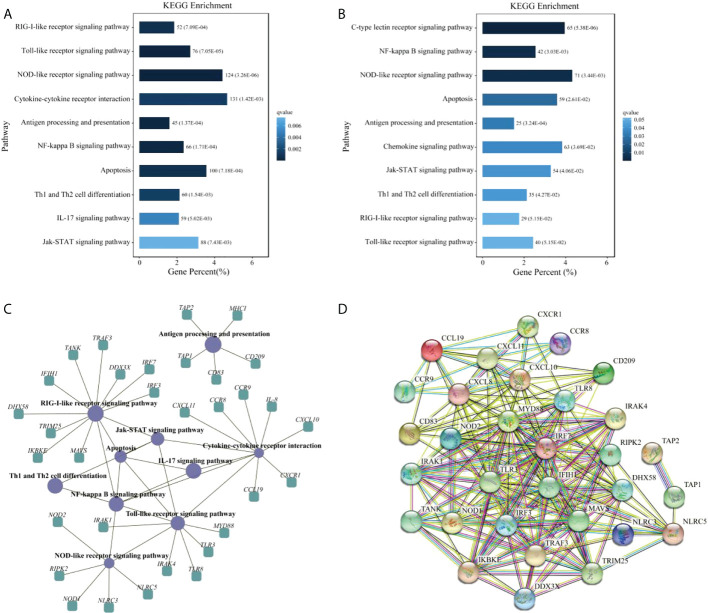
Immune system pathways between C48L and T48L groups. **(A)** Enrichment information of DEGs in immune system pathways. **(B)** Enrichment information of targets of DEMs in immune system pathways. **(C)** Enrichment of key immune-related DEGs in corresponding pathways and interaction of immune system pathways. **(D)** Protein-protein interaction network of immune-related genes obtained in this study.

**Figure 4:**
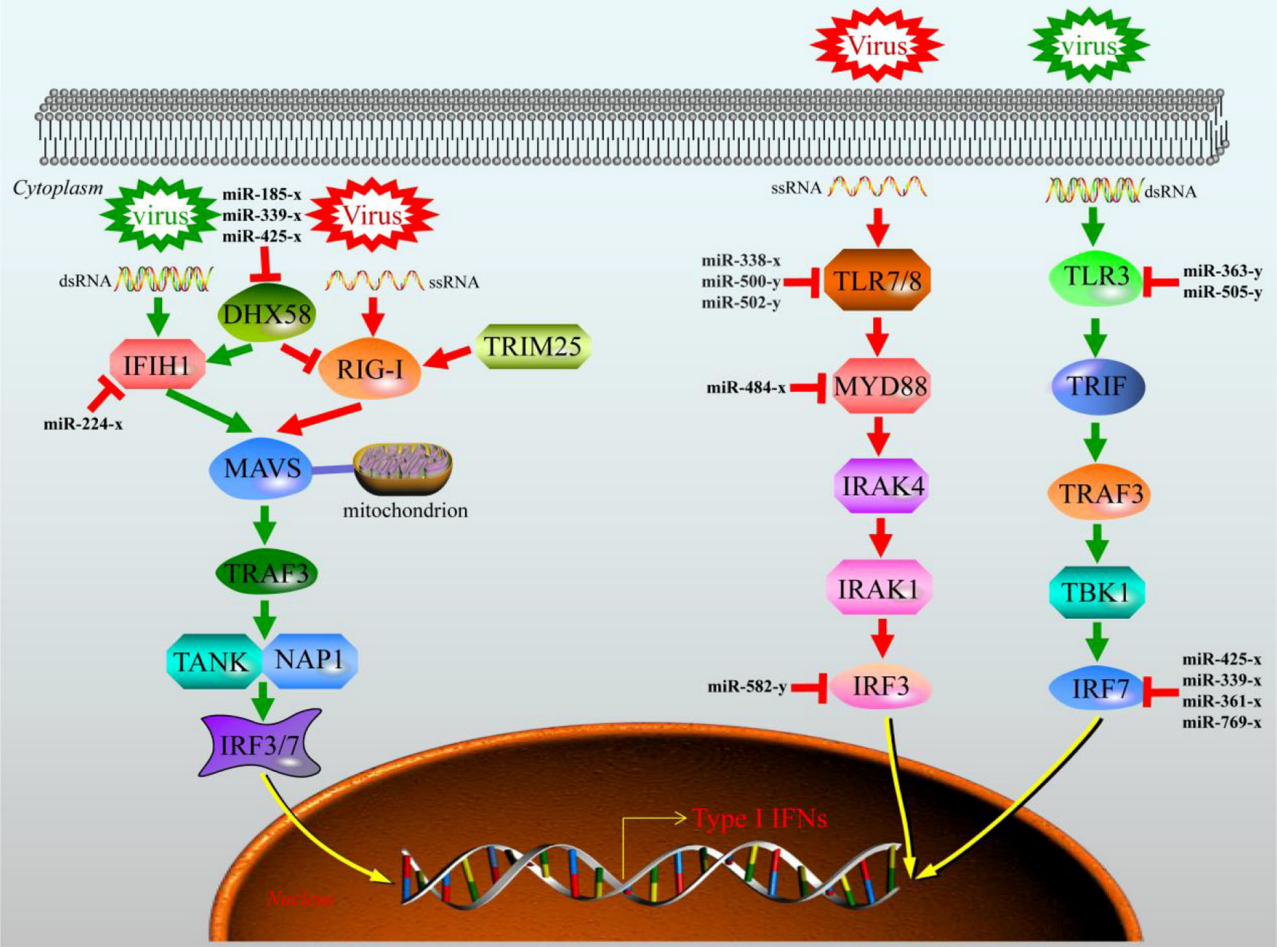
RIG-I like receptor (RLR) and Toll-like receptor (TLR) signaling pathways interaction in signaling against viruses.

To further investigate the function of DEGs, GO enrichment and KEGG pathway analyses were conducted. GO enrichment analysis of DEGs revealed that 48 GO terms were significantly enriched (*q*value < 0.05), in which seven GO terms were closely associated with immunity, including ‘activation of innate immune response’ (GO:0002218), ‘immune system process’ (GO:0002376), ‘immune response’ (GO:0006955), ‘innate immune response’ (GO:0045087), ‘innate immune response-activating signal transduction’ (GO:0002758), ‘regulation of innate immune response’ (GO:0045088), and ‘positive regulation of innate immune response’ (GO:0045089) (**Table S3** and **Figure 2**). We then subjected the DEGs to a pathway enrichment analysis based on the KEGG database and identified multiple immune-related pathways with a *q*value < 0.05, including RLR signaling pathway, TLR signaling pathway, NLR signaling pathway, Jak-STAT signaling pathway, cytokine-cytokine receptor interaction, and antigen processing and presentation (**Table S4** and **Figures 3A, C**), and the interaction of RLR signaling and TLR signaling pathways in the antiviral process is shown in **Figure 4**.

### Identification of DEMs, construction of the miRNA-mRNA network, and GO and KEGG analyses of target genes of DEMs

**Figure 5:**
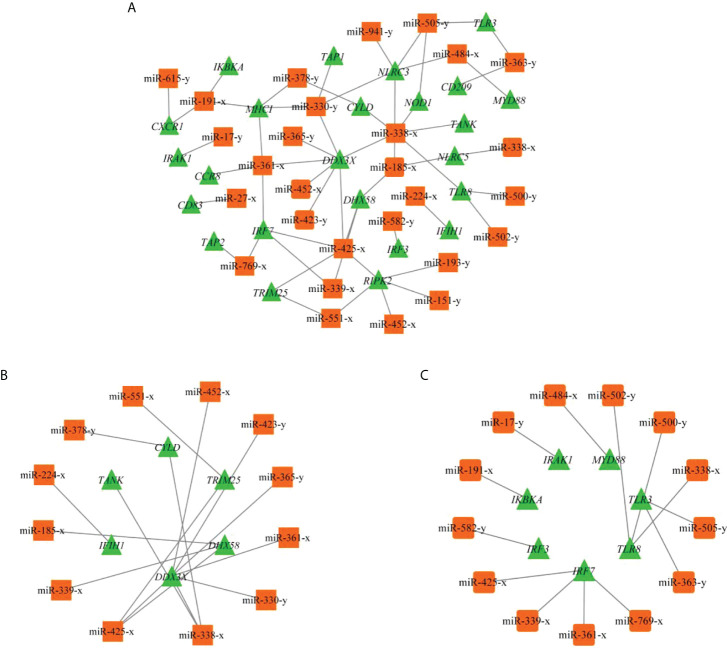
**(A)** miRNA-mRNA regulatory network of immune-related DEGs. **(B)** Subnetwork of immune-related DEGs involved in RLR signaling pathway. **(C)** Subnetwork of immune-related DEGs involved in TLR signaling pathway.

Based on the criteria |Log_2_ fold change | ≥ 1 and *p*value < 0.05, 86 DEMs were identified with 54 known and 32 novel miRNAs, and 34 DEMs were upregulated and 52 were downregulated in T48Ls group (**Table S5** and **Figure S2D**). To explore the role of DEMs, we predicted the target genes that may have regulatory relationship with DEMs. A total of 10,806 negatively correlated miRNA-mRNA pairs were obtained, with the involvement of 86 DEMs and 4042 DEGs (**Table S6**). After analysis, dozens of immune-related miRNA-mRNA pairs were identified (**Figure 5**), and seven miRNAs (miR-425-x, miR-185-x, miR-338-x, miR-330-y, miR-361-x, miR-505-y, and miR-191-x) were found to target at least three key immune-related genes (**Table 4**).

**Table 4:** Representative differentially expressed miRNAs (DEMs) in rainbow trout following infectious hematopoietic necrosis virus (IHNV) challenge.

miRNA-ID	C48Ls_TPM	T48Ls_TPM	Log_2_ (FC)	*p*value	Target gene
miR-425-x	3.01	0.45	–2.73	0.02	*DHX58*/*DDX3X*/*TRIM25*/*IRF7*/*RIPK2*
miR-185-x	3.06	0.12	–4.65	6.95×10^-3^	*DHX58*/*NLRC3*/*NLRC5*
miR-338-x	21.14	8.27	–1.35	4.07×10^-3^	*TANK*/*DDX3X*/*CYLD*/*TLR8*/*NOD1*/*NLRC5*
miR-330-y	1.65	0.01	–7.36	0.01	*DDX3X*/*NLRC3*/*TAP1*/*MHCI*
miR-361-x	6.14	0.99	–2.63	0.01	*DDX3X*/*IRF7*/*CCR8*/*MHCI*
miR-505-y	1.14	0.01	–6.83	0.04	*TLR3*/*NOD1*/*NLRC3*
miR-191-x	176.81	41.16	–2.10	3.96×10^-3^	*IKBKA*/*CXCR1*/*MHCI*

GO and KEGG pathway analyses were performed to filter the key terms and pathways playing vital roles in the process of resisting IHNV with target genes of DEMs. In the GO enrichment analysis, 15 GO terms were enriched with a *q*value < 0.05, including ‘cytokine binding’ (GO: 0019955) (**Table S7**). KEGG enrichment analysis indicated that many pathways were strongly associated with immunity, including NF-kappa B signaling pathway, NLR signaling pathway, antigen processing and presentation, chemokine signaling pathway, Jak-STAT signaling pathway, Th1 and Th2 cell differentiation, RLR signaling pathway, and TLR signaling pathway (**Table S8** and **Figures 3B, C**).

### Expression profile validation

**Figure 6:**
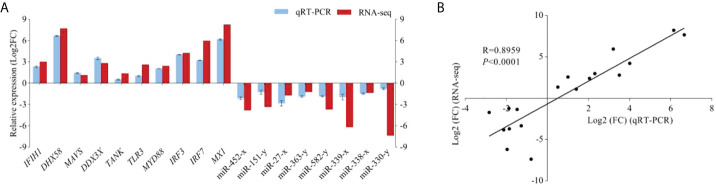
Validation of DEGs and DEMs by qRT-PCR. **(A)** The expression comparison of DEGs and DEMs in RNA-seq and qRT-PCR. Error bars indicate standard deviation. Fold-change of qRT-PCR data represents the ratio of DEGs/DEMs expression values for T48L group vs. C48L group after normalization against *β-actin*/*U6*. **(B)** The Pearson correlation analysis between qRT-PCR and RNA-seq results (R = 0.8959, *P* < 0.0001). The X-axis represents the Log2 (FC) value from qRT-PCR. The Y-axis indicates the Log2 (FC) value from RNA-seq.

To confirm the mRNA and miRNA sequencing data, 10 DEGs and 8 DEMs were selected for qRT-PCR validation. As expected, the results from RNA-seq were in agreement with those from qRT-PCR data in terms of expression trends between C48L and T48L groups (correlation of R = 0.8959), which indicated the high reproducibility and reliability of the RNA-seq results in our study (**Figure 6**).

### Expression patterns analysis of key immune-related genes

**Figure 7:**
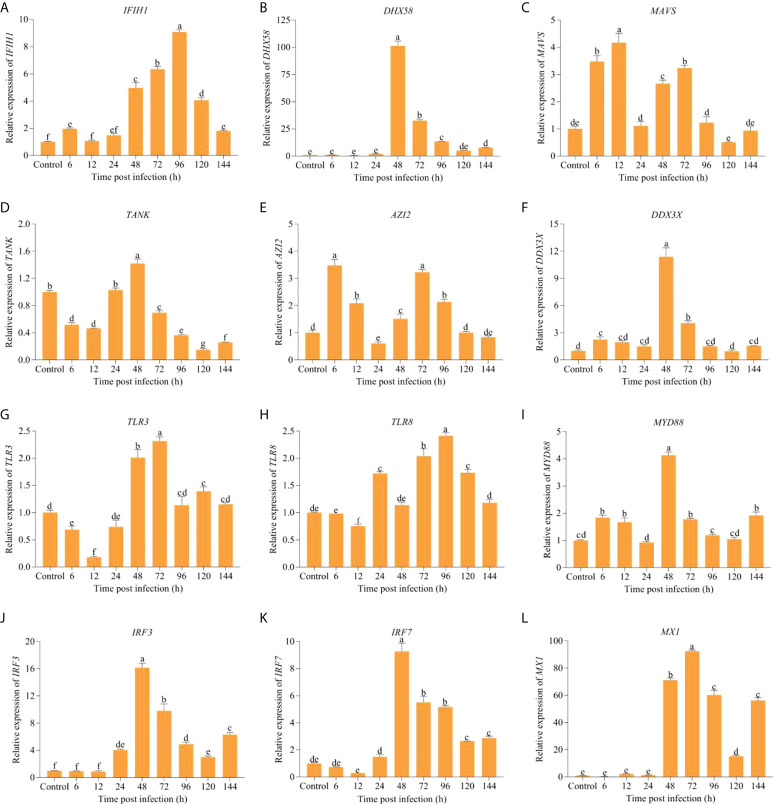
The changes in expression levels of key immune-related DEGs involved in RLR and TLR signaling pathways over different periods after IHNV challenge. **(A)** *IFIH1* (*MDA5*). **(B)** *DHX58* (*LGP2*). **(C)** *MAVS* (*IPS-1*). **(D)** *TANK*. **(E)** *AZI2* (*NAP1*). **(F)** *DDX3X*. **(G)** *TLR3*. **(H)** *TLR8*. **(I)** *MYD88*. **(J)** *IRF3*. **(K)** *IRF7*. **(L)** *MX1*. The different lowercase letters above the bars indicate significant differences (*P* < 0.05).

To further understand the biological roles of immune genes involved in RLR and TLR signaling pathways in anti-IHNV, mRNA levels for *IFIH1*, *DHX58*, *MAVS*, *TANK*, nucleosome assembly protein 1 (*AZI2*/*NAP1*), *DDX3X*, *TLR3*, Toll-like receptor 8 (*TLR8*), *MYD88*, *IRF3*, *IRF7*, and *MX1* were analyzed by qRT-PCR during infection (**Figure 7**). In the RLR signaling pathway, the expression levels of *IFIH1*, *DHX58*, *DDX3X*, *IRF3*, *IRF7*, and *MX1* were upregulated from 24 hpi until 144 hpi compared with the control. Similarly, the mRNA expression of the *MAVS* and *AZI2* was also elevated during most of the post-infection periods, except 24-, 120-, and 144 hpi. However, *TANK* expression was only higher at 24 hpi and 48 hpi than the control. In the TLR signaling pathway, higher expression levels of *TLR3*, *TLR8*, and *MYD88* from 48 hpi to 144 hpi than the control were detected. Combining these two pathways, the expression of *IFIH1* (*P* = 0.00), *DHX58* (*P* = 0.00), *MAVS* (*P* = 0.00), *TANK* (*P* = 4.00 × 10^−3^), *DDX3X* (*P* = 0.00), *TLR3* (*P* = 2.00 × 10^−3^), *MYD88* (*P* = 0.00), *IRF3* (*P* = 2.00 × 10^−3^), *IRF7* (*P* = 5.00 × 10^−3^), and *MX1* (*P* = 0.00) was significantly upregulated at 48 hpi in comparison with the control. In addition, we found that the expression of *DHX58*, *TANK*, *DDX3X*, *MYD88*, *IRF3*, and *IRF7* showed a downward trend after 48 hpi.

### TAP1 was a target of miR-330-y

**Figure 8:**
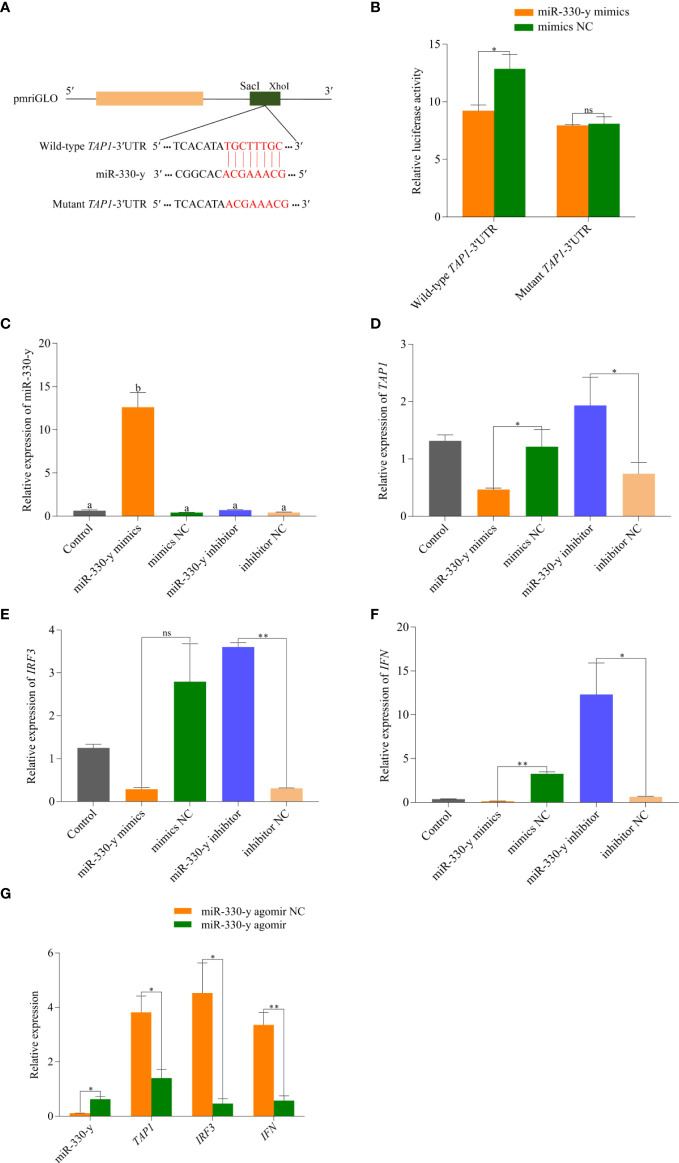
Functional validation of miR-330-y targeting *TAP1 in vitro* and *in vivo*. **(A)** The *TAP1*-3′UTR sequence was inserted into the pmriGLO vector to construct the wild-type and mutant dual luciferase reporter plasmids, respectively. **(B)** The results of dual luciferase reporter assay. **(C)** miR-330-y, **(D)** *TAP1*, **(E)** *IRF3*, **(F)** *IFN* expression levels in rainbow trout liver cells after transfected with miR-330-y mimics or miR-330 inhibitor. **(G)** Effects of miR-330-y agomir on expression of miR-330-y, *TAP1*, *IRF3*, and *IFN in vivo*. The different lowercase letters above the bars indicate significant differences (P < 0.05); *P < 0.05, **P < 0.01; ns, represents no significant difference (P > 0.05).

The bioinformatics software analysis showed that the sites from 2641 bp to 2648 bp (TGCTTTGC) on *TAP1*-3′UTR are most probably able to bind to miR-330-y (**Figure 8A**). The synthesized miR-330-y mimics and NC were than co-transfected into HEK293T cells with the recombinant vector, and the results revealed that miR-330-y mimics significantly reduced the luciferase activity of wild-type *TAP1*-3′UTR (*P* = 0.01), while it showed no effect on activity of luciferase reporter containing mutant *TAP1*-3′UTR (*P* = 0.65) (**Figure 8B**).

### miR-330-y inhibited the expression of TAP1, IRF3, and IFN in liver cells

To verify the function of miR-330-y *in vitro*, miR-330-y mimics and miR-330-y inhibitor were transfected into rainbow trout liver cells. As shown in **Figure 8C–F**, with the increased expression of miR-330-y in miR-330-y mimics group, *TAP1* (*P* = 1.20 × 10^−2^), *IRF3* (*P* = 4.70 × 10^−2^) and *IFN* (*P* = 5.00 × 10^−3^) expression levels were both significantly reduced compared with NC group, and the effects were correspondingly reversed by the miR-330-y inhibitor. The above results suggested that the inhibitory effect of miR-330-y on *TAP1*.

### Effects of miR-330-y on the expression of TAP1, IRF3, and IFN in vivo

As shown in **Figure 8G**, compared with agomir NC group, the miR-330-y expression was markedly upregulated in liver after treated with miR-330-y agomir (*P* = 3.40 × 10^−2^), conversely, the expression levels of *TAP1* (*P* = 2.40 × 10^−2^), *IRF3* (*P* = 2.20 × 10^−2^, and *IFN* (*P* = 5.00 × 10^−3^) were significantly downregualted.

## Discussion

Rainbow trout is a major cold-water species cultured for human consumption around the world, but its development is hindered by various pathogenic viruses in a high-density artificial breeding environment. In recent years, IHNV has led to serious economic losses during rainbow trout farming (20). Mounting evidences have shown that activation and termination of the immune responses are regulated by multiple layers of molecular regulation (21). Thus, it is necessary to make clear the immune defense mechanisms of rainbow trout after the stress of IHNV at the transcriptional and post-transcriptional levels. Herein, we investigated the changes of immune parameters and expression profiles of mRNAs and miRNAs in the liver of rainbow trout following IHNV challenge, and the function of miR-330-y was verified *in vitro* and *in vivo*. These results deepen our understanding of the immune mechanisms in rainbow trout against IHNV.

In the fish body, antibacterial and self-defense antioxidant molecules are required for eliminating various pathogens and maintaining the internal homeostasis, and utilized extensively as relevant biomarkers of the stress response and the general well-being of organisms (22, 23). AKP, ACP, and LZM are important innate immune parameters indicating the stress or disease condition, which participate in the immune activities through non-specific immunity (24). AKP and ACP function as multi-functional enzymes involved in a series of physiological metabolic activities and killing and digesting pathogens (25, 26). LZM, a key defense molecule, plays a crucial role in the defense against exogenous pathogens (27). During bacterial or viral infection, the activity levels of AKP, ACP, and LZM were significantly elevated in pompano (*Trachinotus ovatus*) and silver pomfret (*Pampus argenteus*) (28, 29). In this study, although the ACP activity did not change significantly after IHNV infection compared with the control, both AKP and LZM activity were sensitive to IHNV infection and reached the peak at 48 hpi, suggesting AKP and LZM were activated to protect fish against virus damage. However, significant lower amounts of liver AKP and LZM were observed after 48 hpi. Similar to our study, Chen et al. (25) reported a significant increase in the activity levels of AKP and LZM in the liver and intestine of common carp (*Cyprinus carpio*) on the first day following *Aeromonas hydrophila* infection, and decreased on the fifth and seventh days. Combined, these results indicated that IHNV infection might disrupt the innate immunity in rainbow trout with the proliferation of virus (30, 31).

Under normal physiological conditions, antioxidant defense systems of fish body can scavenge surplus reactive oxygen species (ROS) in time to prevent cell membranes damage and lipid peroxidation (32). Generally speaking, antioxidant enzymes mainly includes T-SOD, CAT, and MDA, which are the most commonly antioxidant indicators in fish (33). T-SOD works in tandem with CAT and it converts harmful ROS into completely harmless compounds (34). MDA content is an essential indicator commonly used to reflect lipid peroxidation (35). During pathogenic attacks, a large amount of ROS accumulation leads to the formation of oxidative stress (36). Studies have shown that *Photobacterium damselae* and *Syndrome coronavirus 2* can significantly increase the activity of T-SOD and CAT in Nile tilapia (*Oreochromis niloticus*) and guppies (*Poecilia reticulate*), respectively (32, 37). Consistent with these studies, a significantly increased T-SOD and CAT were observed at 48 hpi, which were then decreased in the liver of rainbow trout; besides, the content of MDA were significantly reduced throughout, which further illustrated that IHNV may activate antioxidant system in fish and ROS in the liver cannot be completely cleared after 48 hpi. Atencio et al. (38) found that acute oral exposure to *Microcystis* led to decreased T-SOD and CAT activity, and adversely affect the physiological adaptability of Tenca (*Tinca tinca*), eventually causing death. In addition, aminotransferases, ALT and AST enzymatic activities are also as key parameters for evaluating liver functions of fish and related to amino acid metabolism (39, 40). Significantly higher amounts of ALT at 48 hpi and significantly declined levels of both ALT and AST after 48 hpi may be separately related to breakdown of free amino acids for additional energy production for maintenance of homeostasis and liver damage (41).

Once the rhabdoviruses invade physical barriers of the host, the pathogen associated molecular patterns (PAMPs) such as viral G or RNA are monitored by cellular pattern recognition receptors (PRRs), including RLRs and TLRs (2). Recognition of PAMPs leads to trigger intracellular signaling cascades that work towards eliminating the pathogens by producing type I interferon (IFN-I), inflammatory cytokines, and other antiviral factors (42). Currently, there is compelling evidence that IHNV and other novirhabdoviral infections in salmonids can be inhibited by IFN-mediated immune response (43). In the liver samples between CL48m and T48Lm groups, many of the DEGs fell into GO terms and KEGG pathways that were associated with the immune system, suggesting immune response strengthens to maintain the function of liver cells under conditions of IHNV challenge. Among these, it is worth noting that 52 and 76 DEGs fell into RLR and TLR signaling pathway. For RLRs, numerous genes were upregulated after IHNV infection, including *IFIH1*, *DHX58*, and signal mediators (*MAVS*, *TRAF3*, *TANK*, *IRF3*, and *IRF7*). There were also many upregulated genes in the TLRs, such as *TLR3*, *TLR8*, and signal mediators (*MYD88*, *IRAK4*, *IKBKE*, and *IRAK1*). *TLR3*/*MDA5* and *TLR8* act as dsRNA and ssRNA sensor, respectively, and upregulation of their expression contributes to the transcriptional activation of *IFN-I* genes *via* IRF3/7 phosphorylation (44, 45). The upregulation of these genes is similar to results of virus infection studies in Atlantic cod (*Gadus morhua*) and miiuy croker (*Miichthys miiuy*) (46, 47). Actually, IFN-I do not act directly on viral components by themselves, but activate JAK-STAT signaling pathway to induce the anti-viral genes expression, such as *MX1* and *VIG-1*/*2* (48, 49). As expected, *MX1* and *VIG-2* were significantly upregulated in T48Lm group. More importantly, expression levels of *IFIH1*, *DHX58*, *TLR3*, *TLR8*, *IFR3*, *IRF7*, and *MX1* were all higher after 48 hpi (including 48 hpi) than in control, implying that 48 hpi may be a key time point for rainbow trout to against IHNV infection. Taken together, these results suggested that RLR and TLR pathways are important in pattern recognition receptors systems against IHNV infection.

Another PRR is the NLR signaling pathway, which has key function in bacterial and viral immune responses in fish (50). The expression levels related to this pathway were increased in T48Lm group, involving *NOD1*, *NOD2*, *RIPK2*, *NLRC3*, and *NLRC5*. Activation of *NOD1* and *NOD2* induce the production of IFN-I and pro-inflammatory cytokines *via* directly interacting with *MAVS* (51, 52). In zebrafish (*Danio rerio*), overexpression of *NOD2* can result in an increased expression of genes involved in antiviral response, such as *IFIH1*, *RIPK2*, *MAVS*, and *IFN-I*, and these results were similar to previous observations in rainbow trout (53, 54). On the contrary, *NLRC3* and *NLRC5* have been reported to negatively regulate the production of IFN-I and pro-inflammatory cytokines, which can prevent sustained inflammation that may cause damage to self (45). A similar pattern of expression was for *NLRC3* and *NLRC5* in channel catfish (*Ictalurus punctatus*), in which the genes expression was induced significantly in liver by hemorrhage reovirus infection (55). These reports and our present results suggested that *NLRC3* and *NLRC5* play an essential role in protecting hosts against IHNV.

Chemokines are secreted by a range of cell types, and they can recruit immune cells to migrate to the sites of injury or infection and thereby exert immune function (56). The critical antiviral properties of chemokines have been shown in a wide range of fish, such as reovirus infected crucian carp, scale drop disease virus infected barramundi (*Lates calcarifer*), and viral hemorrhagic septicemia virus infected olive flounders (*Paralichthys olivaceus*) (57–59). In the transcriptome data, we found four chemokines: *IL-8*, *CXCL10*, *CXCL11*, and *CCL19*, and three chemokine receptors *CXCR1*, *CCR8*, and *CCR9*, all of which were significantly upregulated in the T48Lm group. *IL-8* is the most significant chemokine for neutrophils by binding to *CXCR1* receptor (60). *CXCL10*, *CXCL11*, and *CCL19* serve as important chemoattractants recruiting cytotoxic lymphocytes (CTLs), natural killer (NK) cells, macrophages, and CD8^+^ T cells (61, 62). In addition, several key DEGs associated with antigen presentation were observed to be significantly upregulated in infected samples, including *CD209*, *CD83*, *TAP1*, *TAP2*, and *MHCI*. *CD209* and *CD83* are known as dendritic cell (a crucial member of antigen presenting cells) markers, and a previous study in zebrafish found that CD209 blockade resulted in visible suppression of T cell activation, IgM production, and bacterial vaccination-elicited immunoprotection (63). *TAP1*, *TAP2*, and *MHCI* are responsible for presenting antigenic peptides from the cytosol to T cells (64). Furthermore, upregulation of *TAP1* is conducive to the IFN-I production *via* activating the IRF3 signaling transduction (65). All of these molecules involved in antigen presentation have been proved to be related to the antiviral responses of host whether in fish or mammals (66, 67). Together with the transcriptome data obtained in this study and indicated that in addition to the innate immune response, the specific immune response against IHNV may occur locally at the infected site.

It is known that miRNAs can downregulate host cellular immune-related genes by base-pairing with target 3′UTR of mRNA, thereby directly regulate viral infection process (13). In teleost fish, a large quantity of miRNAs have been reported to be involved in various signaling pathways in the innate immune system, including PRRs, and showed that one miRNA can target multiple immune-related genes, indicating the importance of miRNAs in host antiviral responses (68). Accordingly, understanding the regulatory mechanism of miRNAs in host-pathogen interactions contributes to open new avenues for identifying biomarkers and improving the efficacy of therapies against IHNV in rainbow trout. In the present study, 10,806 negatively corrected miRNA-mRNA pairs were identified. Of these, seven miRNAs were found to target at least three key immune-related genes discussed above, including miR-425-x, miR-185-x, miR-338-x, miR-330-y, miR-361-x, miR-505-y, and miR-191-x. A prior study showed that involvement of miR-425 in inflammatory responses by acting with miR-191 can reduce gastric cancer cell proliferation and migration (69). Huang et al. demonstrated that miR-185 can restrain hepatitis C virus replication (70). miR-330 was involved in the regulatory mechanism of NLRP3 inflammasome, and can inhibit the development of liver cancer, gastric cancer, and osteosarcoma (71, 72). As those reports, we also correspondingly found that when up regulated the expression level of miR-330-y by its mimics or agomir *in vitro* and *in vivo*, the expression level of *TAP1* was significantly downregulated, meanwhile a marked decrease in mRNA levels of *IRF3* and *IFN* were detected, indicating the production change of IFN-I during rainbow trout antiviral process is likely to be influenced by miR-330-y along with its target *TAP1*. Thus, it is reasonable that these miRNAs associated with immune-related pathways may play invaluable roles in immune response to IHNV infection. However, functions of other miRNAs in rainbow trout resistance to IHNV remain to be further studied.

## Conclusion

This study systematically elucidated the changes in immune parameters and immune-related genes expression, and the miRNA-mRNA interaction in rainbow trout liver infected by IHNV. We found that APK, ALT, CAT, and T-SOD activities and MDA content were reached peak at 48 hpi, followed by a decrease. According to the results of mRNA and miRNA sequencing, dozens of miRNA-mRNA pairs involved in RLR signaling pathway, TLR signaling pathway, NLR signaling pathway, cytokine-cytokine receptor interaction, and antigen processing and presentation were identified. The expression levels of most key immune-related genes enriched in RLR and TLR signaling pathways increased after 24 hpi compared with the control. Furthermore, the function of miR-330-y was verified *in vitro* and *in vivo*. The results increase our understanding of the molecular mechanisms in rainbow trout against IHNV, and lay a foundation for further studying the regulatory functions of miRNAs on key immune-related genes.

## Data availability statement

The datasets presented in this study can be found in online repositories. The names of the repository/repositories and accession number(s) can be found below: NCBI under accession number GSE205742.

## Ethics statement

The animal study was reviewed and approved by Gansu Agricultural University.

## Author contributions

SW organized and wrote the manuscript. JH designed the experiment. YL and ZL modified the manuscript. ML and LZ performed bioinformatics data analysis. All authors contributed to the article and approved the submitted version.

## Funding

This study was supported by the Supporting Funds for Youth Mentor of Gansu Agricultural University (GAU-QDFC-2018-09), Fuxi Youth Talent Training Program of Gansu Agricultural University (Gaufx-02Y08), and Fostering Foundation for the Excellent Ph.D. Dissertation of Gansu Agricultural University (YB2022001).

## Conflict of interest

The authors declare that the research was conducted in the absence of any commercial or financial relationships that could be construed as a potential conflict of interest.

## Publisher’s note

All claims expressed in this article are solely those of the authors and do not necessarily represent those of their affiliated organizations, or those of the publisher, the editors and the reviewers. Any product that may be evaluated in this article, or claim that may be made by its manufacturer, is not guaranteed or endorsed by the publisher.

## Supplementary material

Supplementary Table 1 The sequence of primers used in this study.

Supplementary Table 2 Differentially expressed genes (DEGs) between C48Lm and T48Lm groups.

Supplementary Table 3 Gene Ontology (GO) enrichment results of DEGs.

Supplementary Table 4 Kyoto Encyclopedia of Genes and Genomes (KEGG) enrichment results of DEGs.

Supplementary Table 5 Differentially expressed miRNAs (DEMs) between C48Ls and T48Ls groups.

Supplementary Table 6 Negatively correlated miRNA-mRNA pairs formed by 86 DEMs and 4042 DEGs.

Supplementary Table 7 Gene Ontology (GO) enrichment results of the target genes.

Supplementary Table 8 Kyoto Encyclopedia of Genes and Genomes (KEGG) enrichment results of the target genes.

Supplementary Figure 1
**(A)** Heatmap of Pearson correlation between samples of control (C48Lm) and infected (T48Lm) groups. **(B)** Length distribution of all expressed miRNAs in the six libraries. **(C)** Number of known and novel miRNAs of each sample. **(D)** Distribution of gene expression abundance of each sample.

Supplementary Figure 2 Characteristics of mRNA and miRNA expression levels between C48L and T48L groups. **(A)** Volcano plot of distribution trends for differentially expressed genes (DEGs). **(B, C)** Heatmaps of expression levels for all DEGs and immune-related genes. **(D)** Scatter map of differentially expressed miRNAs (DEMs).
