# Supplementary figures and images for: Integrated analysis of immune parameters, miRNA-mRNA interaction, and immune genes expression in the liver of rainbow trout following infectious hematopoietic necrosis virus infection
Source: Front Immunol. 2022 Sep 2;13:970321. doi: 10.3389/fimmu.2022.970321 (PMC9479325; doi:10.3389/fimmu.2022.970321)

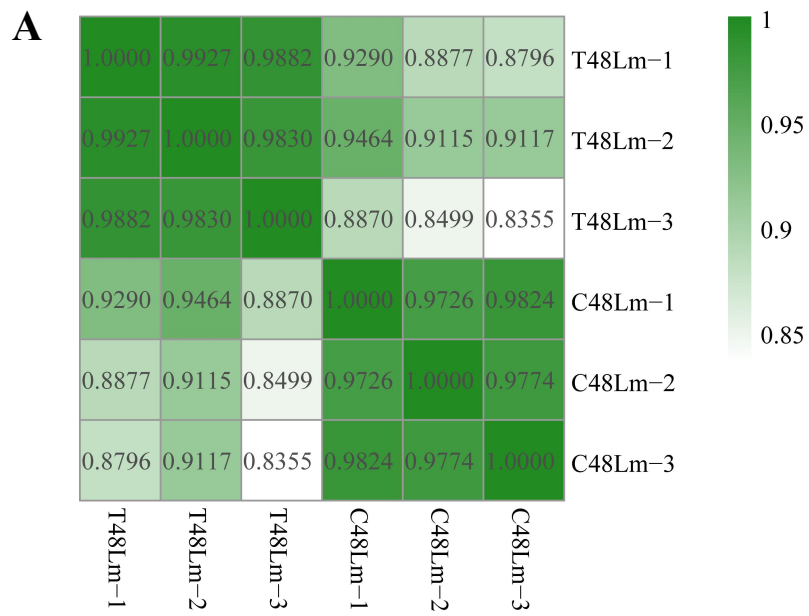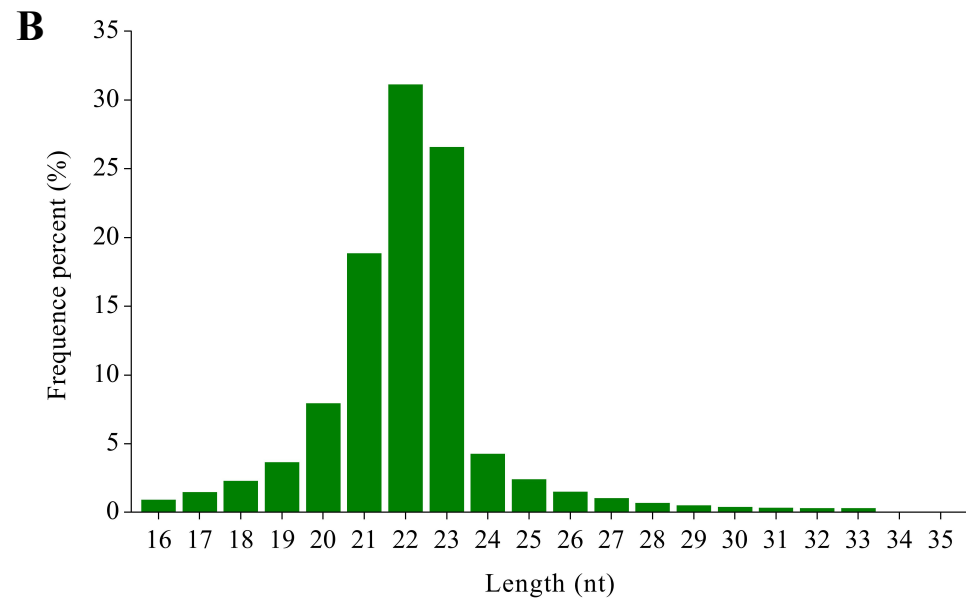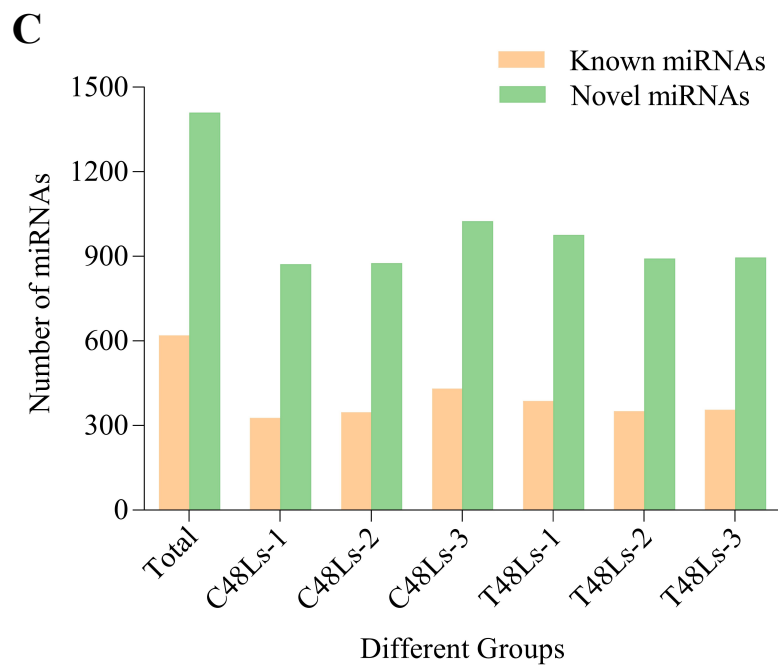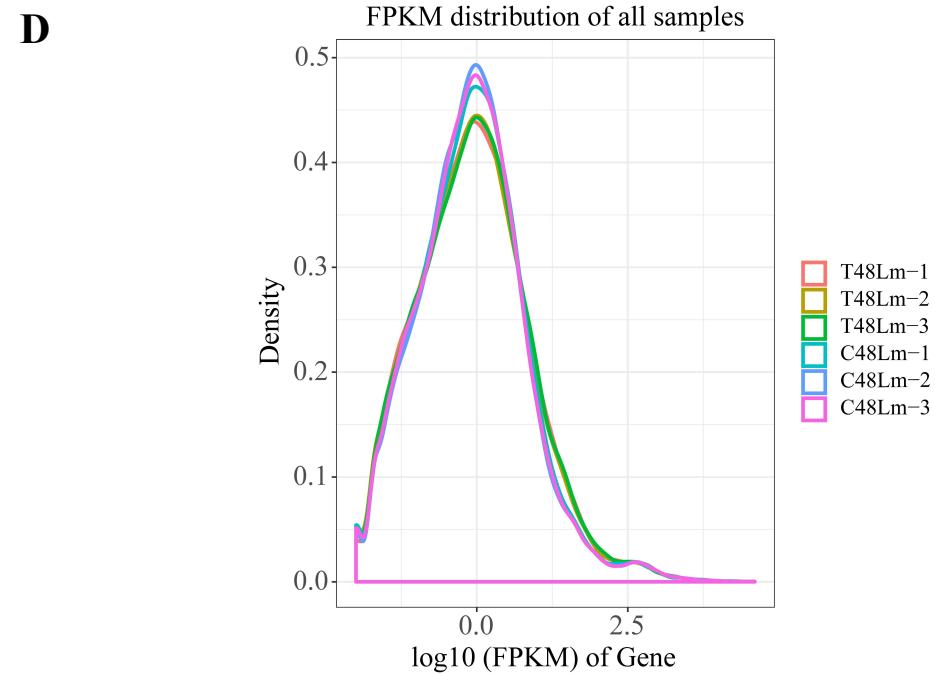

Supplement: Supplementary Figure 1 — (A) Heatmap of Pearson correlation between samples of control (C48Lm) and infected (T48Lm) groups. (B) Length distribution of all expressed miRNAs in the six libraries. (C) Number of known and novel miRNAs of each sample. (D) Distribution of gene expression abundance of each sample. [file DataSheet_1.pdf]

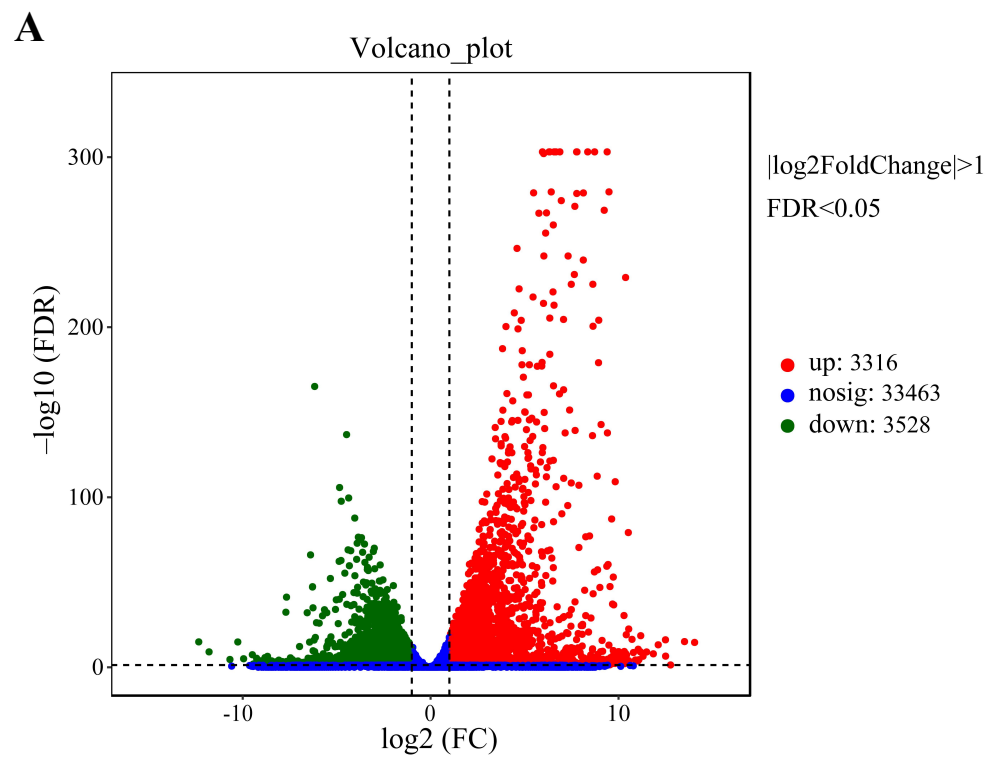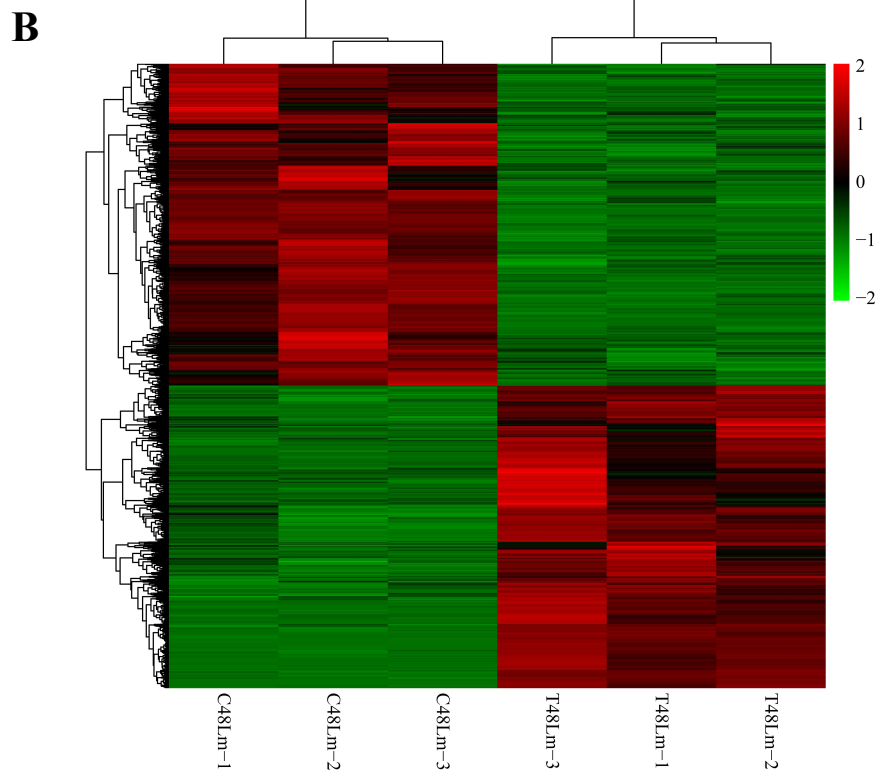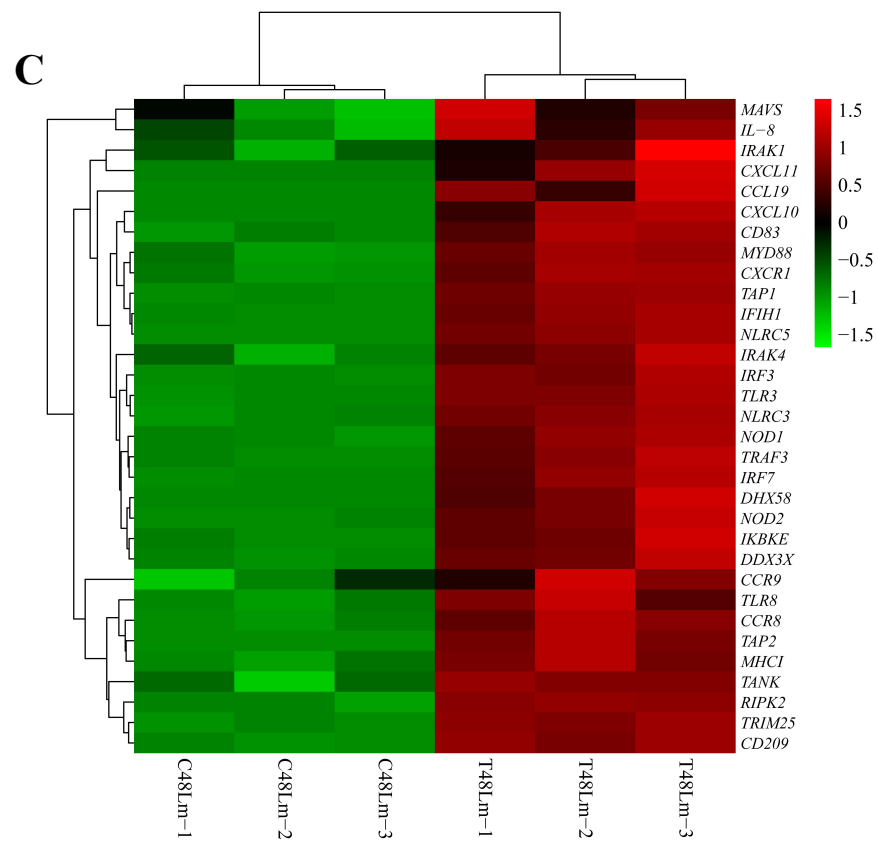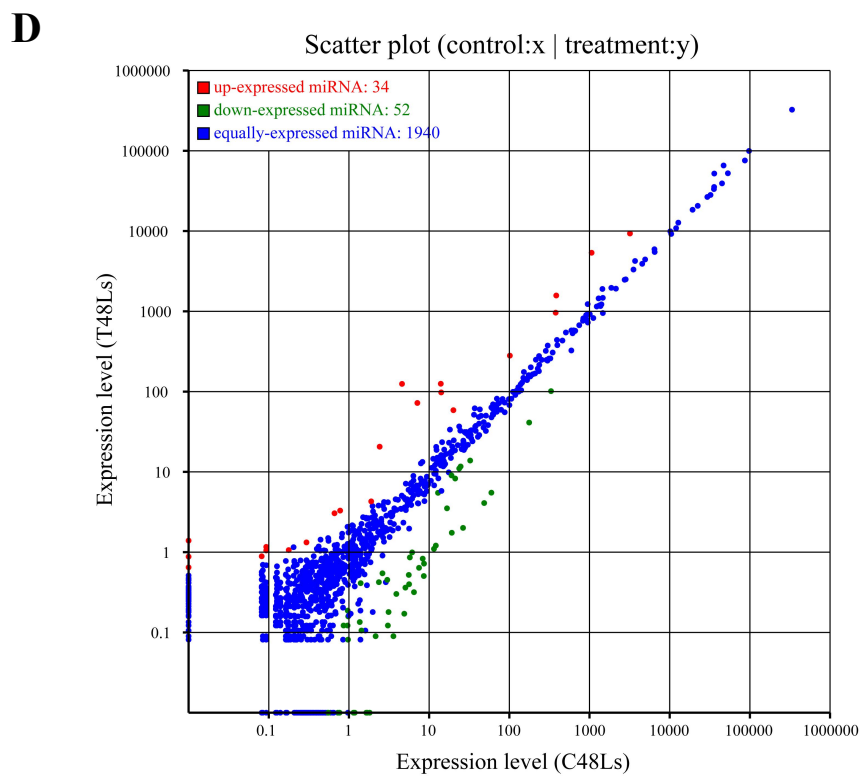

Supplement: Supplementary Figure 2 — Characteristics of mRNA and miRNA expression levels between C48L and T48L groups. (A) Volcano plot of distribution trends for differentially expressed genes (DEGs). (B, C) Heatmaps of expression levels for all DEGs and immune-related genes. (D) Scatter map of differentially expressed miRNAs (DEMs). [file DataSheet_2.pdf]
